# Stability and recovery issues concerning chondroitin sulfate disaccharide analysis

**DOI:** 10.1007/s00216-021-03152-7

**Published:** 2021-01-27

**Authors:** Gábor Tóth, Domonkos Pál, Károly Vékey, László Drahos, Lilla Turiák

**Affiliations:** 1 MS Proteomics Research Group, Research Centre for Natural Sciences, Eötvös Loránd Research Network, Budapest, 1117 Hungary; 2 Department of Inorganic and Analytical Chemistry, Budapest University of Technology and Economics, Budapest, 1111 Hungary

**Keywords:** Chondroitin-sulfate, Glycosaminoglycan, High-performance liquid chromatography, Mass spectrometry, Stability, Recovery

## Abstract

Chondroitin sulfate (CS) is a widely studied class of glycosaminoglycans, responsible for diverse biological functions. Structural analysis of CS is generally based on disaccharide analysis. Sample preparation is a key analytical issue in this case. However, a detailed study on the stability and recovery of CS-derived species has been lacking so far. We have found that for solvent exchange, in general, vacuum evaporation (SpeedVac) is much preferable than lyophilization. Moreover, in the case of aqueous solutions, higher recovery was experienced than in solutions with high organic solvent content. Storage of the resulting disaccharide mixture in typical HPLC injection solvents is also critical; decomposition starts after 12 h at 4 °C; therefore, the mixtures should not be kept in the sample tray of an automatic injector for a long time. The study, therefore, lays down suggestions on proper sample preparation and measurement conditions for biologically derived chondroitin sulfate species.

**Supplementary Information:**

The online version contains supplementary material available at 10.1007/s00216-021-03152-7.

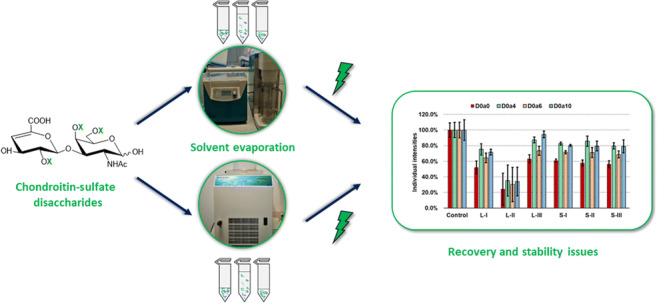

## Introduction

Chondroitin sulfate (CS) is a class of glycosaminoglycans (GAGs), composed of characteristic disaccharide building blocks. These disaccharides are built up by one N-acetylgalactosamine (GalNAc) and one glucuronic acid unit. The backbone is synthesized in the Golgi apparatus, followed by sulfation carried out by various sulfotransferases [1]. Sulfation can occur at various positions, the most common case is GalNAc 4-OH, 6-OH, and glucuronic acid 2-OH sulfation [2]. These sulfations may or may not be present in any of the above-mentioned positions providing versatile structure and function to the CS chain. The GAG chains have been proven to be responsible for cellular signaling and recognition, concerning the size and sulfation pattern of the respective chains [3–5]. Alterations in the sulfation motifs may be descriptive of various diseases, e.g., cancer progression [6, 7].

Structural characterization of CS by instrumental analytical tools is usually performed by analyzing their oligosaccharide or disaccharide composition after enzymatic digestion (e.g., Chondroitinase ABC) or chemical degradation (deaminative cleavage with nitrous acid) [8, 9]. Nowadays, the most common type of analysis is HPLC-MS investigation of disaccharide building blocks. Chondroitinase ABC digestion results in Δ^4,5^-unsaturated disaccharides listed in Table 1. By disaccharide analysis, an average sulfation pattern can be obtained which is descriptive of sulfation motifs of the original molecules.

**Table 1:**
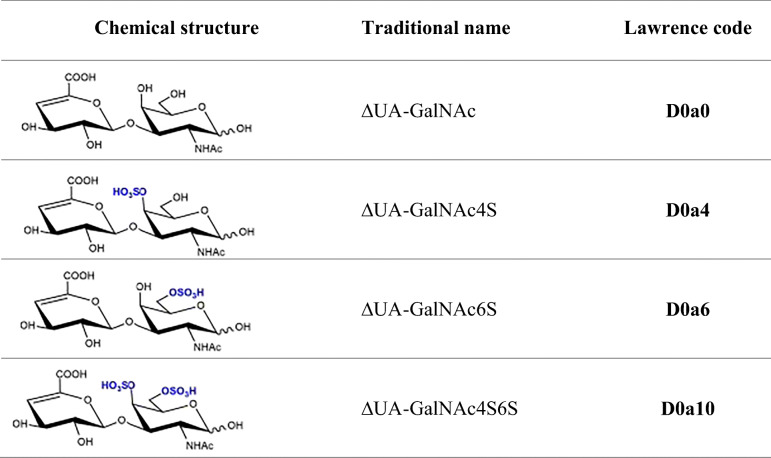
Structure and nomenclature of the CS disaccharides investigated

Usually, prior to analysis, the samples undergo different sample preparation (cell lysis, enzymatic digestion, enrichment) and cleanup procedures including solid phase extraction (SPE) and solvent evaporation. The SPE methods are well-studied; however, to our knowledge, there has been no literature for the (quite concerning) recovery issues during sample evaporation steps.

Various chromatographic methods (with UV or MS detection) have been reported to analyze sulfation patterns of CS disaccharides [10, 11]. Some examples are the following: reversed-phase chromatography with derivatization or ion-pairing [12, 13], size exclusion (SEC) [14], graphitized carbon [15], HILIC [16, 17], or HILIC-WAX [18, 19] chromatography. The most common injection solvents for the abovementioned methods are water, acetonitrile, methanol, and their mixtures with or without buffer salts, in order to match the initial conditions of the chromatographic run. Prior to injection to the HPLC system, the samples can be stored for several hours in an autosampler unit which, according to our previous experience, can cause a substantial incertainty in the measured quantities. The CS disaccharides can decompose via beta-elimination, sulfate loss, and monosaccharide loss [20].

Volpi et al. [20] investigated the stability of CS chains under acidic, neutral, and basic conditions, and Zaia et al. [21] gave information on 4-*O* and 6-*O* sulfated CS disaccharides in 30:70 v/v% methanol:water injection solvent. Our group has previously shown the potential instability of CS disaccharides during storage at 4 °C in the injection solvent 75:25 v/v% acetonitrile:water (10 mM ammonium formate, pH = 4.4) used for their HILIC-WAX chromatographic method [18].

To summarize, variance caused by recovery issues during sample preparation and autosampler storage stability makes up for most of the inconsistency experienced during analysis. However, only a few studies have dealt with recovery and stability of CS disaccharides so far. Thus, in the present work, we performed a detailed analysis on the stability and recovery of CS disaccharides during various sample preparation steps (solvent evaporation with heated vacuum centrifuge and lyophilizer) and storage conditions (water, acetonitrile, methanol, and salt mixtures at 4 °C). This information provides an important background to resolve potential problems and to help design bias-free analytical workflows for this class of compounds.

## Materials and methods

### Chemicals and reagents

The Δ^4,5^-unsaturated chondroitin sulfate disaccharide standards (listed in Table I, “CS disaccharides” hereinafter) were purchased from Iduron (Cheshire, UK). Crystalline ammonium formate, ammonium bicarbonate, Chondroitinase ABC, and formic acid (FA) were purchased from Merck (Budapest, Hungary). LC-MS grade water, acetonitrile, and methanol were purchased from VWR International Ltd. (Debrecen, Hungary).

### Liquid chromatography–mass spectrometry

A Waters® nanoAcquity UPLC system (Waters, Milford, MA, USA) was coupled to a Waters® QTOF Premier™ Mass Spectrometer (Waters, Milford, MA, USA) via ESI source. The analysis was performed with a method previously reported [18]. Briefly, a 250 μm × 10.5 cm capillary column packed with GlycanPac AXH-1 resin (Unicam Plc., Debrecen, Hungary) was used at a temperature of 45 °C with a flow rate of 8 μL/min. Eluent A was 10 mM ammonium formate in 75:25 v/v ACN:water (pH 4.4); eluent B was 65 mM ammonium formate in 75:25 v/v ACN:water (pH 4.4). Starting from 6% B, the eluent ratio changed in 0.5 min to 12% B, and then in 4.5 min to 60% B. MS parameters were the following. Capillary voltage: 2.1 kV; sampling cone: 17 eV; extraction cone: 4 V; the ion guide: 1.5; source temperature: 80 °C; desolvation temperature: 100 °C; cone gas flow: 25 L/h; the desolvation gas flow: 300 L/h; collision energy: 22 eV. The investigated compounds were measured as singly and doubly charged anions and sodium adducts.

### Evaporation study experiments

For determining the effects of the evaporation apparatus and volume, 10 pmol of each analyte was added to the sample, and they were diluted with 10 μL water (S-I and L-I), 100 μL water (S-II and L-II), and 100 μL water/10 mM ammonium bicarbonate (S-III and L-III). Four samples were made for each condition. Samples were then dried down with a Barnstead Genevac miVac heated vacuum centrifuge (55 °C, SpeedVac) or a Scanvac CoolSafe lyophilizer (− 110 °C), respectively, and stored at − 20 °C until further usage.

For determining the effects of the evaporation solvent, 10 pmol of each analyte was added to the sample, and they were diluted in 10 μL of the following solvents: 90:10 v/v ACN:water (A), 75:25 v/v ACN:water (B), 10 mM ammonium formate (pH 4.4) in 75:25 v/v ACN:water (C), 50:50 v/v ACN:water (D), 75:25 v/v MeOH:water (E), and 10 mM ammonium formate (pH 4.4) in 75:25 v/v MeOH:water (F). Three samples were made for each condition. Samples were then dried down with a SpeedVac heated vacuum centrifuge (55 °C, until completely dry) and stored at − 20 °C until further usage.

The dry samples were reconstituted in 10 μL of injection solvent (10 mM ammonium formate in 75:25 v/v ACN:water (pH 4.4)) and were stored in the autosampler unit for a maximum of 2 h at 4 °C. One microliter was injected (1 pmol of each analyte).

### Storage stability experiments

For determining the stability in generally used injection solvents, 10 pmol of each analyte was pooled and dissolved in 10 μL of the following solvents: 90:10 v/v ACN:water (A), 75:25 v/v ACN:water (B), 10 mM ammonium formate (pH 4.4) in 75:25 v/v ACN:water (C), 50:50 v/v ACN:water (D), 75:25 v/v MeOH:water (E), and 10 mM ammonium formate (pH 4.4) in 75:25 v/v MeOH:water (F). The samples were stored in Eppendorf tubes at 4 °C for the following timeframes: 4 h, 8 h, 12 h, 16 h, 20 h, 24 h. Four sample parallels were prepared for each condition. After the determined incubation time, samples were immediately dried down with a SpeedVac heated vacuum centrifuge (55 °C, 15 min) and stored at − 20 °C until further usage.

The dry samples were reconstituted in 10 μL of injection solvent (10 mM ammonium formate in 75:25 v/v ACN:water (pH 4.4)) and were put in the autosampler unit (4 °C) just before injection. One microliter was injected (1 pmol of each analyte).

## Results and discussion

The stability and recovery of CS disaccharides is a crucial but neglected area of HPLC-MS analysis. For this reason, we decided to examine the bottlenecks of sample preparation and analysis in the following respects: solvent evaporation and storage in the autosampler.

**Fig. 1:**
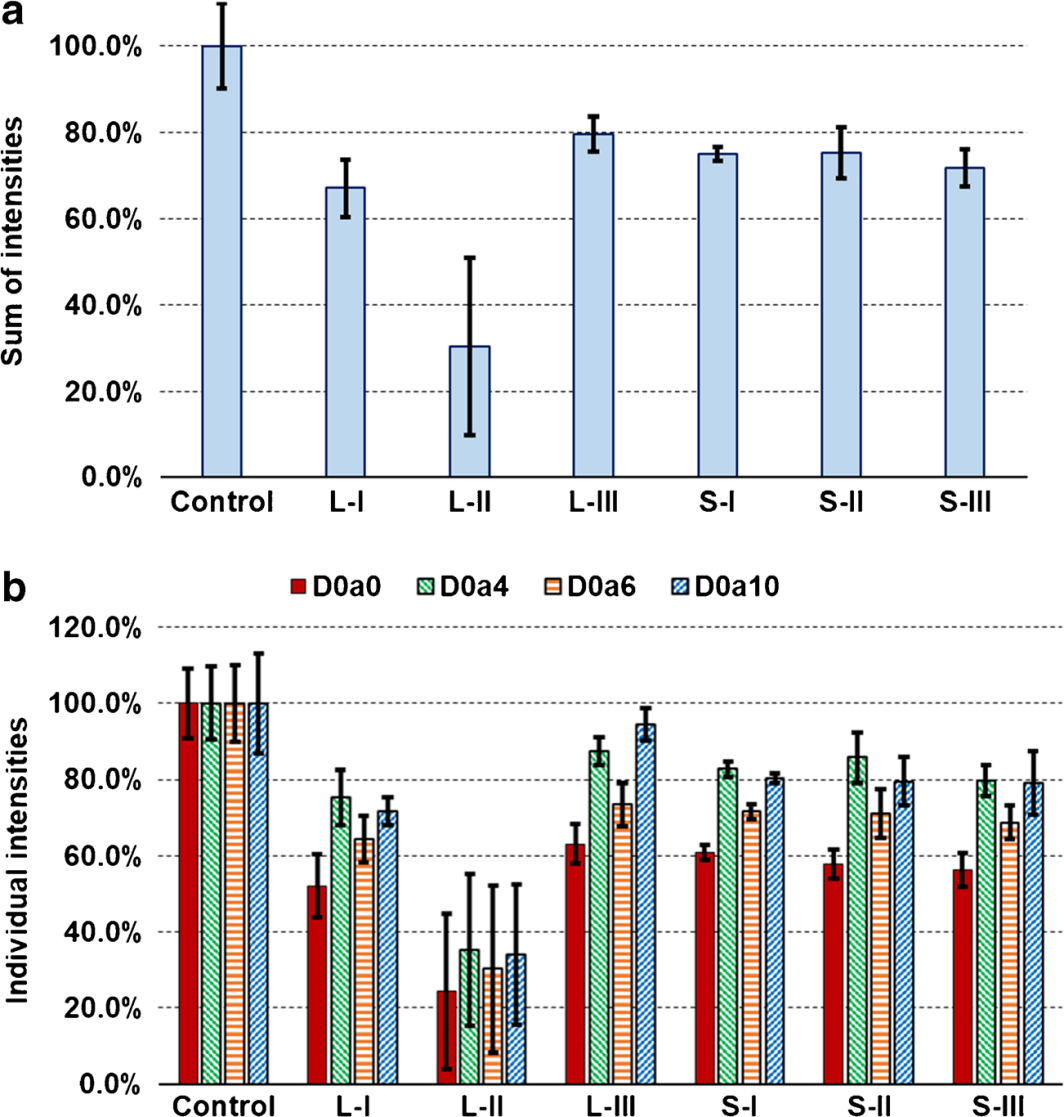
Recovery of CS disaccharides during solvent evaporation methods. **a** Summed intensity of the four CS disaccharides, relative to the non-evaporated control sample. **b** Intensities of individual disaccharides, relative to the non-evaporated control sample. (L: lyophilization; S: SpeedVac; I: 10 μL water; II: 100 μL water; III: 100 μL water with 100 mM ammonium bicarbonate)

First, we compared two generally used solvent evaporation methods: SpeedVac heated vacuum centrifuge (S) and lyophilization (L) using a mixture of CS disaccharide standards. Both techniques were used to evaporate the solvent from 10 μL (S-I and L-I) and 100 μL aqueous samples (S-II and L-II) and 100 μL aqueous samples with 100 mM ammonium bicarbonate (S-III and L-III) and compared to a non-evaporated control sample (C). As is seen in Fig. 1, a remarkable difference could be observed between the two methods. Using SpeedVac (S), 70–80% of the total disaccharide amount was recovered with a similar average from the three kinds of samples (Fig. 1a). However, when we used lyophilizer (L), the sample volume and composition strongly determined the degree of recovery: the small sample volume and the ammonium bicarbonate content resulted in similar recovery values to the SpeedVac evaporation (67–80%), but evaporating from 100 μL pure water caused a major sample loss (ca. 30% recovery on average). The sulfation pattern was modified by the two methods in a similar way (Fig. 1b). D0a0 provided the smallest recovery under these conditions, followed by D0a6, while D0a4 and D0a10 showed similarly good values. It is also important to mention that the use of lyophilization caused a much higher deviation in the peak areas, being the largest from 100 μL pure water (RSD: 37–44%). The additional variance in the samples dried with SpeedVac was comparable with the variance of the HPLC-MS method, moreover, evaporating from 10 μL aqueous sample (S-I) gave outstanding results (inside the variance of HPLC-MS, RSD < 5%). Therefore, we advise the use of SpeedVac evaporation from the smallest volume possible for CS disaccharide analysis. This method was used in our further experiments as well.

**Fig. 2:**
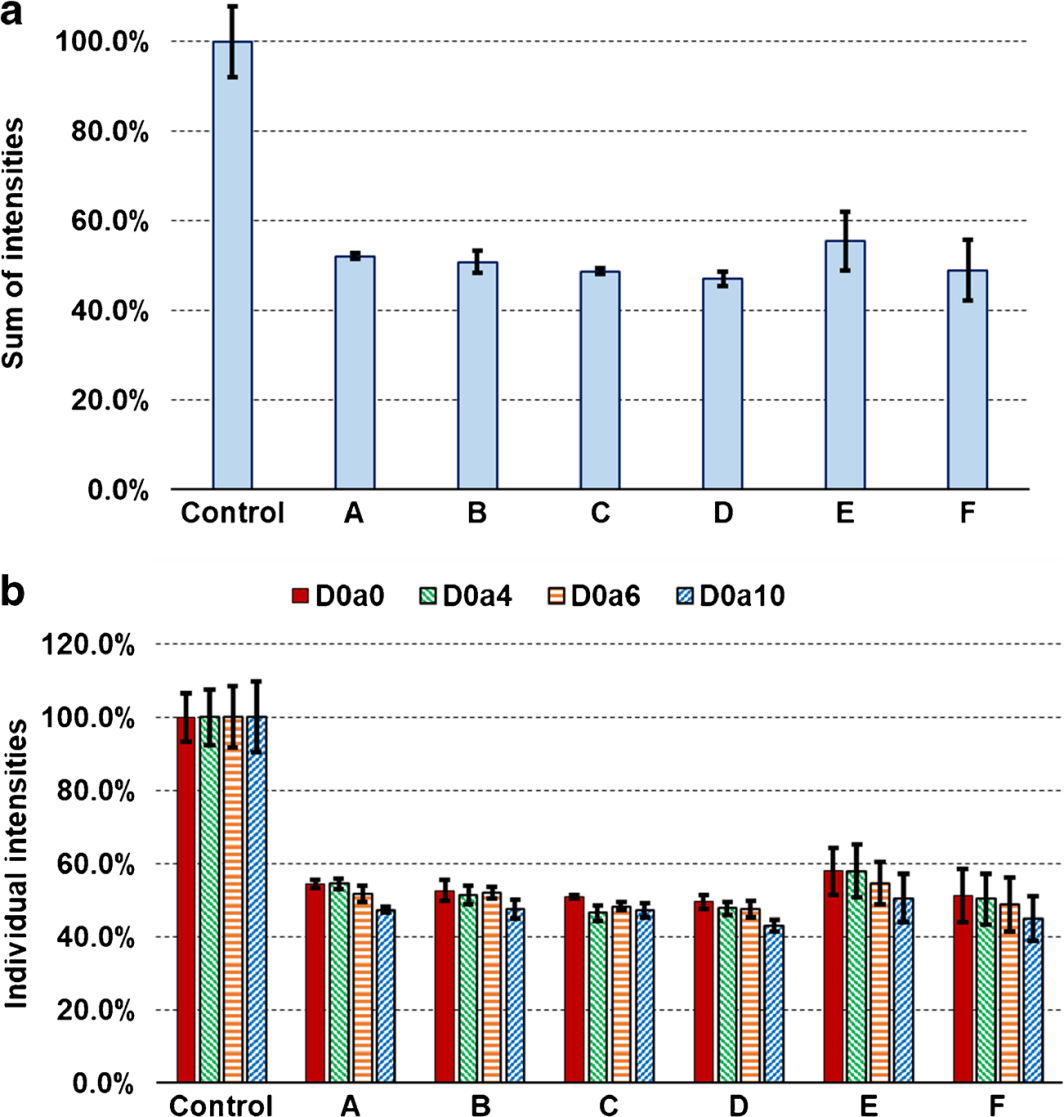
Recovery of CS disaccharides during SpeedVac solvent evaporation from various solvent mixtures. **a** Summed intensity of the four CS disaccharides, relative to the control sample. **b** Intensities of individual disaccharides, relative to the non-evaporated control sample. (A: 90:10 v/v ACN:water; B: 75:25 v/v ACN:water; C: 10 mM ammonium formate (pH 4.4) in 75:25 v/v ACN:water; D: 50:50 v/v ACN:water; E: 75:25 v/v MeOH:water; F: 10 mM ammonium formate (pH 4.4) in 75:25 v/v MeOH:water)

Second, we explored the effects of different solvent mixtures on SpeedVac evaporation recovery from 10 μL volumes of the following solvents: 90:10 v/v ACN:water (A), 75:25 v/v ACN:water (B), 10 mM ammonium formate (pH 4.4) in 75:25 v/v ACN:water (C), 50:50 v/v ACN:water (D), 75:25 v/v MeOH:water (E), and 10 mM ammonium formate (pH 4.4) in 75:25 v/v MeOH:water (F). Contrary to what was observed for pure water in the previous section, the mixtures containing organic solvents caused major sample loss (50% on average, Fig. 2a). The sample loss was not affected by either the type or the ratio of the organic solvent. The sulfation pattern suffered only a minor change: the D0a10 disaccharide provided 8–11% less recovery in all solvents, except for the solvent C (Fig. 2b), but the change was statistically significant only in the case of solvents A and D. It can also be concluded that the methanol-containing solvents (solvents E and F) caused larger variance, but this was comparable with the variance of the HPLC-MS analysis.

Third, we addressed the stability of the analytes under autosampler conditions in most of the generally used injection solvent mixtures. We determined the stability of CS disaccharides after 4 h, 8 h, 12 h, 16 h, 20 h, and 24 h incubation at 4 °C in the following solvents: 90:10 v/v ACN:water (A), 75:25 v/v ACN:water (B), 10 mM ammonium formate (pH 4.4) in 75:25 v/v ACN:water (C), 50:50 v/v ACN:water (D), 75:25 v/v MeOH:water (E), and 10 mM ammonium formate (pH 4.4) in 75:25 v/v MeOH:water (F). Since we compared generally used injection solvents, we did not aim to deeply investigate the effects of various pH values on the rate of decomposition. Since it was shown that the SpeedVac evaporation caused major sample loss, all the control samples for this study were prepared in the respective solvent and evaporated at zero incubation time.

**Fig. 3:**
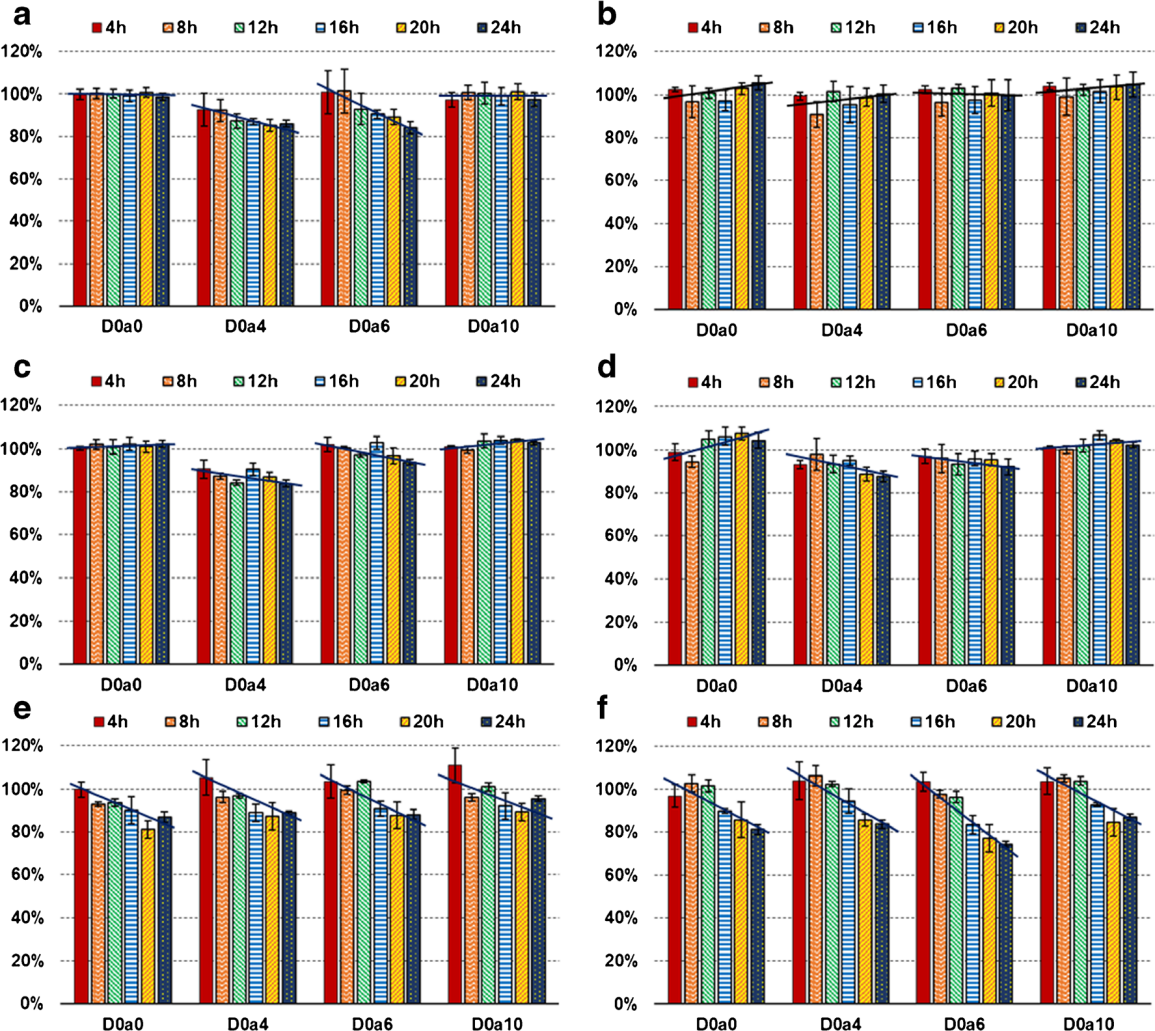
Stability of CS disaccharides during 4 °C storage in various solvent mixtures. Intensities of individual disaccharides are represented, relative to the control sample. **a** 90:10 v/v ACN:water; **b** 75:25 v/v ACN:water; **c** 10 mM ammonium formate (pH 4.4) in 75:25 v/v ACN:water; **d** 50:50 v/v ACN:water; **e** 75:25 v/v MeOH:water; **f** 10 mM ammonium formate (pH 4.4) in 75:25 v/v MeOH:water)

In acetonitrile-based solvents (Fig. 3a–d), the non-sulfated (D0a0) and disulfated (D0a10) components showed outstanding stability during the time of investigation. However, the monosulfated CS disaccharides (D0a4 and D0a6) showed a decreasing trend in three of the four ACN-based solvents, the exception being solvent B, 75:25 v/v ACN:water (Fig. 3b). The rate of decomposition was significant (measured value under 90%) for D0a4 in solvents A and C and for D0a6 in solvent A (Fig. 3a and c). Thus, the storage of CS disaccharide samples is not recommended for more than 12 h in solvents A and C (due to the decomposition of the D0a4 disaccharide), while bias-free 24 h storage of samples can be acquired with solvents B and D.

In methanol-based solvents (Fig. 3e and f), all the components showed significant decomposition over time with a similar rate of decomposition. In 75:25 v/v MeOH:water, the measured values of all the components fell below 90% at the 16 h measurement point which is in correlation with the data formerly reported on monosulfated components by Zaia et al. [21]. Using the solvent 10 mM ammonium formate (pH 4.4) in 75:25 v/v MeOH:water, over 90% of D0a0, D0a4, and D0a10 components were conserved for 16 h, but the D0a6 disaccharides decomposed with a slightly higher rate. Since the instability of only one analyte can compromise the results, the storage in MeOH-based solvents is not recommended for over 12 h.

Finally, we characterized the main decomposition products derived from each disaccharide. We discovered that the sulfated CS disaccharides may undergo sulfuric acid elimination to a high degree, in accordance with former NMR studies [20]. A peak for N-acetyl galactosamine was also observed which is derived from the decomposition into monosaccharide units. The decomposition products were observed in only minor amounts during the evaporation study and in higher quantites during the solvent stability studies. This implies that during evaporation, non-specific interaction with the tube walls accounts for the sample loss, and during sample storage, decomposition takes place. For details, please see Supplementary information (ESM).

In conclusion, the recovery and stability of CS disaccharides has been studied from two aspects: the influence of solvent evaporation during sample preparation and stability of the sample solution during autosampler racking. For the evaporation of solvents, we recommend the use of SpeedVac, from the smallest volume possible, since it results in small variance and conservation of the sulfation pattern. Nevertheless, sample loss as much as 20% is expected in the case of aqueous solutions, while from high organic solvent–containing mixtures, losses may increase to 50% due to inspecific interations occurring between the disaccharides and the tube walls. Note that although a significant amount of the sample may be lost, this does not influence the sulfation pattern appreciably. Disaccharides may easily decompose during storage in the autosampler, even if it is cooled to 4 °C. In the case of MeOH-based solvents, autosampler storage is not recommended for over 12 h, since all CS disaccharides decompose at a high rate. In the case of ACN-based solvents, the decomposition rate of the monosulfated disaccharides (D0a4 and D0a6) presents the worst problem. Storage is best in 50:50 v/v and 75:25 v/v ACN:water, as it results in a bias-free analysis even after 24 h.

## Supplementary information

ESM 1 (PDF 195 kb)

## Data Availability

Not applicable.
