# Supplementary material for: Stability and recovery issues concerning chondroitin sulfate disaccharide analysis
Source: Anal Bioanal Chem. 2021 Jan 27;413(7):1779–85. doi: 10.1007/s00216-021-03152-7 (PMC7925331; doi:10.1007/s00216-021-03152-7)
Supplement: Supplementary file 1 — (PDF 195 kb) [file 216_2021_3152_MOESM1_ESM.pdf]

Stability and recovery issues concerning chondroitin-sulfate disaccharide analysis

Supplementary material to the article published in
Analytical and Bioanalytical Chemistry.

Gábor Tóth[a,b], Domonkos Pál[a,b], Károly Vékey[a], László Drahos[a], Lilla Turiák[a,*]

[a]MS Proteomics Research Group, Research Centre for Natural Sciences, H-1117 Budapest, Hungary

[b]Department of Inorganic and Analytical Chemistry, Budapest University of Technology and Economics, H-1111 Budapest, Hungary

ORCID IDs:
Gábor Tóth: 0000-0003-1428-7906; Károly Vékey: 0000-0002-8131-3628; László Drahos: 0000-0001-9589-6652; Lilla Turiák: 0000-0002-2139-8156

Key words: chondroitin-sulfate; glycosaminoglycan; high-performance liquid chromatography; mass spectrometry; stability; recovery

***Corresponding author**

Lilla Turiák
Research Centre for Natural Sciences
Magyar tudósok körútja 2., H-1117, Budapest, Hungary
Email: turiak.lilla@ttk.hu
Tel: +36 1 382 6516

We aimed to discover the potential decomposition products formed in solution during storage.

We used two different chromatographic methods: the standard gradient (see 4.2 chapter in the main body) and an altered gradient for spreading the early eluting decomposition products. MS conditions were the same. Only those species were considered decomposition products that had different retention times from the parent species using both methods. (Same retention time means identical chromatographic behavior, thus most probably the structure is the same and the ion is fromed by decomposition in the mass spectrometer.)

The altered chromatographic conditions were the following. A 250 µm x 10.5 cm self-packed GlycanPac AXH-1 capillary column was used, the column temperature was set to 45°C, and the flow rate was adjusted to 8 µL/min. Eluent A was 10 mM ammonium formate in 75:25 v/v ACN:water (pH 4.4); Eluent B was 65 mM ammonium formate in 75:25 v/v ACN:water (pH 4.4). Starting from 0% B, the eluent ratio changed in 4 min to 2% B, and then in 2 minutes to 60% B. As a washing step, the composition was held elevated to 100% B and held for 4 minutes and it was followed by a 5-minute-long equilibration at the initial composition.

We discovered that the CS disaccharides can suffer a major sulfuric acid elimination, in accordance with former NMR studies (1). A peak for N-acetyl galactosamine was also observed which is derived from the decomposition into monosaccharide units (Fig. S1).

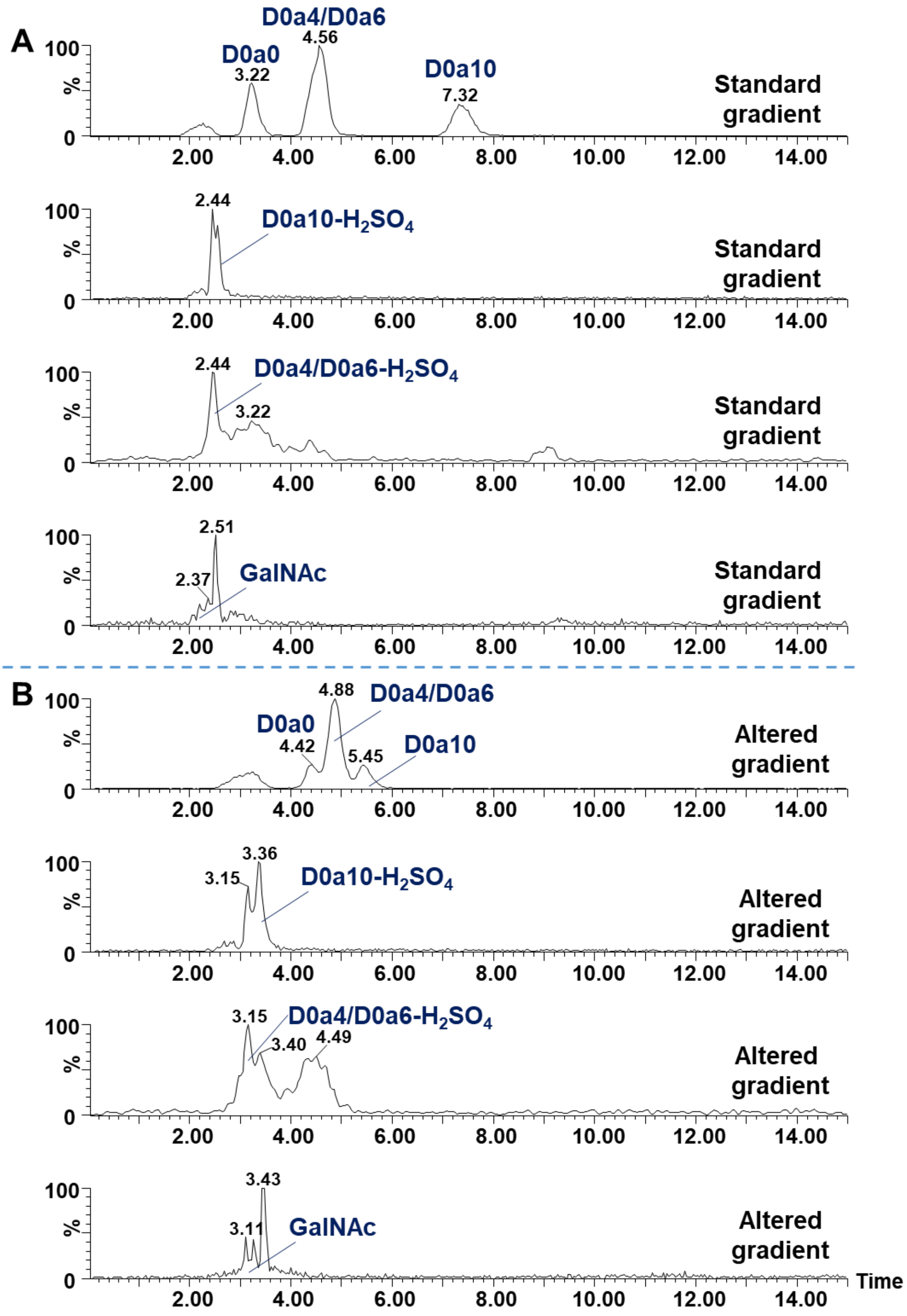

Fig. S1 Extracted ion chromatograms (EICs) of CS disaccharides and their most abundant decomposition products. A: EICs using the standard gradient, B: EICs using an altered gradient for spreading the early eluting components

1. Volpi N, Mucci A, Schenetti L. Stability studies of chondroitin sulfate. Carbohydrate research. 1999;315(3-4):345-9.
